# Anticancer Mechanism of Curcumin on Human Glioblastoma

**DOI:** 10.3390/nu13030950

**Published:** 2021-03-16

**Authors:** Shu Chyi Wong, Muhamad Noor Alfarizal Kamarudin, Rakesh Naidu

**Affiliations:** 1 Jeffrey Cheah School of Medicine and Health Sciences, Monash University Malaysia, Jalan Lagoon Selatan, Bandar Sunway, Selangor Darul Ehsan 47500, Malaysia; wsc_1997@hotmail.com; 2 Brain Research Institute Monash Sunway (BRIMS), Jeffrey Cheah School of Medicine and Health Sciences, Monash University Malaysia, Jalan Lagoon Selatan, Bandar Sunway, Selangor Darul Ehsan 47500, Malaysia; muhamadnoor.alfarizal@monash.edu

**Keywords:** curcumin, glioblastoma, anticancer, molecular signaling mechanism

## Abstract

Glioblastoma (GBM) is the most malignant brain tumor and accounts for most adult brain tumors. Current available treatment options for GBM are multimodal, which include surgical resection, radiation, and chemotherapy. Despite the significant advances in diagnostic and therapeutic approaches, GBM remains largely resistant to treatment, with a poor median survival rate between 12 and 18 months. With increasing drug resistance, the introduction of phytochemicals into current GBM treatment has become a potential strategy to combat GBM. Phytochemicals possess multifarious bioactivities with multitarget sites and comparatively marginal toxicity. Among them, curcumin is the most studied compound described as a potential anticancer agent due to its multi-targeted signaling/molecular pathways properties. Curcumin possesses the ability to modulate the core pathways involved in GBM cell proliferation, apoptosis, cell cycle arrest, autophagy, paraptosis, oxidative stress, and tumor cell motility. This review discusses curcumin’s anticancer mechanism through modulation of Rb, p53, MAPK, P13K/Akt, JAK/STAT, Shh, and NF-κB pathways, which are commonly involved and dysregulated in preclinical and clinical GBM models. In addition, limitation issues such as bioavailability, pharmacokinetics perspectives strategies, and clinical trials were discussed.

## 1. Introduction

Brain tumors can be classified into grade I and II (benign, low-grade), grade III (malignant, high-grade) such as anaplastic astrocytoma, and grade IV (highly aggressive and malignant) such as glioblastoma (GBM) [1]. GBM is the most common and aggressive form of malignant primary adult brain tumor [2]. In the United States alone, the annual age-adjusted incidence of GBM is 3.22 per 100,000 persons based on registry data from 2012 to 2016 [2]. Based on the 2016 WHO classification of the central nervous system tumors, GBM is classified as a grade IV diffuse glioma. GBM is further classified into isocitrate dehydrogenase-wildtype (IDH-wildtype), IDH-mutant, and not otherwise specified (NOS) [3]. IDH-wildtype or primary (de novo) GBM accounts for 90% of the total proportion of GBM cases [3]. The IDH-mutant or secondary GBM, which may arise from a lower grade diffuse glioma, only accounts for about 10% of the total GBM cases [3]. Primary GBM is more common in elderly patients (median age of 62 years), while secondary GBM preferentially arises in younger patients (median age of 44 years) [3].

The standard care for newly diagnosed GBM patients is surgical resection, followed by radiotherapy (60Gy in 30 fractions) with concurrent oral administration of temozolomide (TMZ), followed by six cycles of adjuvant [4]. Additionally, monoclonal antibody bevacizumab and other alkylating agents such as carmustine, lomustine, nimustine, and fotemustine are used in GBM treatment [4]. Unfortunately, these treatments often prove ineffective, given the poor prognosis outcomes of GBM with a five-year survival rate under 10% and a median survival rate of around 12 to 18 months [2,5]. The high infiltration degree of GBM often causes surgical resection incapable of fully resecting the GBM tumor, leaving the residual presence of microscopic foci [6,7]. Moreover, the GBM tumors often develop chemo- and radio-resistance with the formation of glioma stem cells, leading to GBM recurrence [8]. In TMZ-resistant GBM tumors, numerous molecular pathways such as nuclear factor kappa light chain enhancer of activated B cells (NF-κB), p53, and JAK-STAT are found to be commonly dysregulated [8]. In addition, several clinical complications such as pancytopenia, pyrexia, wound healing complications, multi-organ failure, or even death are observed following the chemo-radiation and immunotherapy treatment [8,9,10].

Thus, in recent years, scientists have been focusing on phytochemicals as potential therapeutic agents in cancer management to minimize drug toxicity and side effects. Flavonoids represent the most common and widely distributed phytochemicals in fruits and vegetables. Various flavonoids such as tannins, quinones, stilbenes, and curcuminoids possess antioxidant, anti-inflammatory, antiviral, antimutagenic, and, most importantly, anticancer properties [11,12]. Among them, curcuminoids (especially curcumin) have been gaining immense attention because of its anticarcinogenic, antitumor, antioxidant, and anti-inflammatory actions [13,14,15]. Curcuminoids are a family of active compounds found in the turmeric rhizome (*Curcuma longa*), an Indian spice commonly used in cooking. Natural curcuminoids are composed of curcumin, bisdemethoxycurcumin, and desmethoxycurcumin in a proportion of 77:3:17 [16]. Among them, curcumin is the most abundant compound and has been widely studied as a potential therapeutic agent in chronic diseases, such as neurodegenerative, cardiovascular, pulmonary, metabolic, and autoimmune diseases [17]. For instance, curcumin was able to restore oxidative stress and DNA methyltransferase (DNMT) functions against diabetic retinopathy [15]. Curcumin also acts as a wound healing promoting agent by facilitating collagen synthesis and fibroblast migration [18]. Several pre-clinical and clinical studies also reported its anticancer effects in colorectal cancer [19,20], pancreatic cancer [21], lung cancer [22], and GBM [23]. Curcumin can modulate multiple cellular signaling pathways and molecular targets involved in GBM tumor growth, migration, invasion, cell death, and proliferation [24,25,26,27]. Retinoblastoma (Rb), p53, MAP kinase (MAPK), P13K/Akt, JAK/STAT, sonic hedgehog (Shh), and NF-κB pathways are the most common targeted dysregulated pathways found in GBM and modulated by curcumin [28,29,30,31,32,33,34]. Moreover, curcumin is highly lipophilic and able to cross the blood–brain barrier (BBB) [35,36].

To date, numerous review studies have suggested curcumin as a potential drug for GBM. However, a greater focus on curcumin’s anticancer potential in molecular signaling pathways that are commonly dysregulated in GBM is needed to provide a more comprehensive understanding of its therapeutic effects. This review includes the initial until recent pre-clinical and clinical studies of curcumin’s mechanisms of action in modulating several molecular pathways such as Rb, p53, MAPK, P13K/Akt, JAK/STAT, Shh, and NF-κB pathways. This review paper also discusses curcumin’s related issues such as low bioavailability, pharmacokinetics, and the perspective strategies to overcome these issues.

## 2. Dysregulated Signaling Pathways Associated with GBM Pathogenesis

Almost all GBMs are found to have dysregulated Rb, p53, JAK/STAT, MAPK, P13K/Akt, Shh, and NF-κB pathways. Thus, the following section discusses the mechanisms of action modulated by curcumin via these molecular signaling pathways involved in GBM cell proliferation, apoptosis, cell cycle arrest, autophagy, paraptosis, oxidative stress, and cell motility. The reported observation of in vitro and in vivo studies of curcumin against GBM are summarized in Table 1.

### 2.1. Retinoblastoma (RB) Pathway

The RB pathway plays a central role in cell proliferation by regulating the cell cycle [56]. This pathway mainly consists of five components, which are CDKN2A/p16Ink4a, cyclin D1, cyclin-dependent protein kinases (cdk4/6), RB-family of pocket proteins (RB, p107, p103), and E2F [57,58]. CDKN2A/p16 is a negative regulator that competes with cyclin D1 to bind to and inhibit the activity of CDK4/6. This, in turn, can induce cell cycle arrest at the G1/S phase by inhibiting phosphorylation of RB protein by the cyclin D1/cdk4/6 complex. The unphosphorylated RB protein binds to E2F protein to inhibit the activation of E2F-regulated gene expression, thereby inhibiting cell cycle progression, DNA replication, and nucleotide biosynthesis [57].

Many of the important components of this pathway are frequently altered in many cancer cells, including GBM [59]. According to The Cancer Genome Atlas (TCGA) pilot project, most of the GBM acquired mutations include homozygous deletion or mutation of *CDKN2A/p16* and *RB1*, and amplification of *CDK4*, *CDK6*, and *cyclin D*, which are associated with the RB signaling pathway [60]. *RB* promoter methylation and gene silencing are found in GBMs and are more frequently reported in secondary GBMs than primary GBMs [61]. The inhibition of the RB pathway via silencing/suppression of its component proteins increases etoposide-induced DNA double strand breaks, p53 activation, and TMZ-induced GBM apoptosis [62,63,64,65]. Additionally, the inhibition of cyclin D1 can downregulate P-glycoprotein (pgp) expression, which may help to overcome chemoresistance in GBMs [63]. Thus, the RB signaling pathway is an important drug targeted pathway to improve GBM prognosis and patient outcomes.

**Figure 1:**
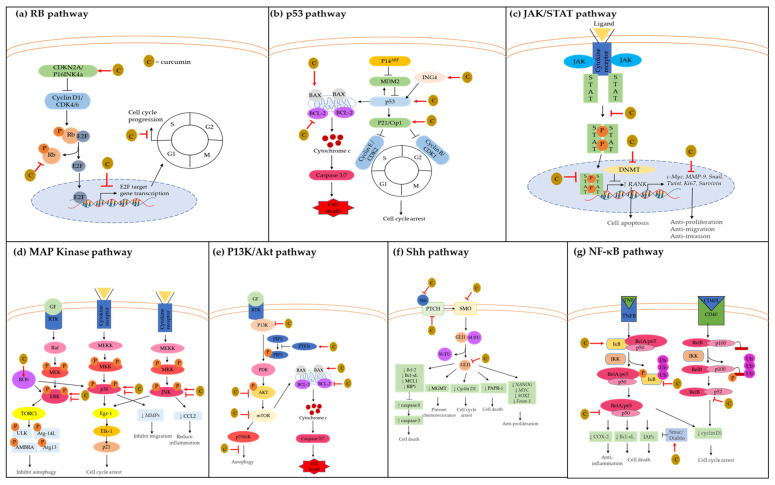
Mode of curcumin actions as anti-cancer agent on the key molecular targets in GBM. Curcumin possesses anti-cancer properties by inhibiting signaling pathways and their downstream molecular targets; (**a**) retinoblastoma (RB) pathway; (**b**) p53 pathway; (**c**) JAK1,2/STAT3 pathway; (**d**) MAP kinase pathway; (**e**) P13K/Akt pathway; (**f**) Shh pathway; (**g**) NF-κB pathway. Molecular targets and signaling pathways that are upregulated and downregulated by curcumin are noted by using → and ┤, respectively.

As shown in Figure 1a, curcumin inhibits the RB signaling pathway by increasing the negative regulator CDKN2A/p16Ink4a activity, which then suppresses the phosphorylation of RB protein. To date, Chin-Cheng Su and colleagues demonstrated that curcumin significantly inhibited the RB pathway in DBTRG glial cells in a time- and concentration-dependent manner [28]. In this study, curcumin treatment upregulated CDKN2A/p16 and downregulated the phosphorylated RB protein. It has been shown that CDKN2A/p16 protein compete with cyclin D1 to bind to CDK4/6 protein, which then inhibits phosphorylation of RB protein. Unphosphorylated RB protein could not dissociate from its repressor E2F to permit transcription of G1 genes for proceeding from G1 to S phase.

### 2.2. P53 Pathway

P53 is a tumor suppressor protein that can activate cell cycle arrest or induce cell apoptosis to prevent damaged cells from further dividing and growing [66]. Following DNA damage, p53 is activated to induce transcription of p21Waf/Cip1, a cyclin-dependent kinase inhibitor [58]. This P21Waf/Cip1 protein can induce G1/S and G2/M arrest by binding to and inhibiting the activity of Cdc2, cyclin-CDK2, -CDK1, and CDK4/6 complexes. This allows the damaged cells to undergo DNA repair prior to mitosis. The p53 activity can be inhibited by MDM2, the transcription of which is induced by TP53 through a negative feedback loop [67]. However, P14ARF, which is located in part of the CDKN2A locus, can bind to and inhibit MDM2 from binding to the N-terminal transactivation domain of TP53 [58,68]. On the other hand, activated p53 protein can activate the pro-apoptotic BH3-only members of the Bcl-2 protein family [69]. These pro-apoptotic proteins bind and inhibit the pro-survival Bcl-2 proteins to initiate the pro-apoptotic multi-BH domain members of the Bcl-2 family, such as BAX and BAK, to induce cell apoptosis.

Dysregulation of p53 pathway in GBM is mostly due to *TP53* mutation, amplification of *MDM2*, or loss of expression of CDKN2A-p14ARF [70]. The TCGA project demonstrated that the p53 signaling pathway is altered in most GBM samples with the association of *TP53* mutation or homozygous deletion, *P14ARF* deletions, and amplification of *MDM2* and *MDM4* [60]. According to WHO, the *TP53* mutation is more commonly seen in secondary GBM and is higher in proportion to primary GBM [3]. The clinical study showed that most of the sample cells (from GBM patients with age around 56) were Bcl-2 positive, and most of the Bcl-2 positive cloned cells acquired chemoresistance [71]. Thus, various strategies have been developed to target the p53 pathway, such as inhibition of pro-survival genes or MDM2/p53 interaction, degradation of mutant p53, and restoration of wildtype p53 [72,73,74].

As shown in Figure 1b, curcumin upregulates the expression of p53, p21, and ING4 and increases the BAX:BCL2 ratio to induce cell cycle arrest and apoptosis. Curcumin upregulates the expression of p53 and p21 in a time- and concentration-dependent manner, which then induces G2/M arrest in DBTRG cells [28]. Curcumin induces p53 activity by upregulating p21Waf/Cip1 and ING4 protein expression [37]. The upregulation of ING4 expression can increase p53 acetylation at Lys-382 and protein stability [75,76]. The acetylation of p53 inhibits its interaction with MDM2, which eventually induces cell cycle arrest and apoptosis, as observed in U251 cells [37]. In a different study, curcumin enhances paclitaxel (PTX) activity in C6 cells by increasing TP53 and p21 gene expression. In response to the increase of TP53 and p21 gene expression, the cell population in the G0/G1 phase increases while the cell population in the S phase decreases, indicating G0/G1 phase arrest [38]. Moreover, curcumin suppresses A172 cell viability by inducing paraptosis through the regulation of genes associated with the endoplasmic reticulum (ER) stress response [40]. Interestingly, in this study, interaction network analysis (with IPA software) revealed that the altered levels of ER-related miRNAs interact with p53-BCL2 pathways. Thus, it was suggested that the p53-BCL2 pathway might be involved in curcumin anticancer mechanisms. Additionally, curcumin potentiates the cytotoxic and apoptosis-inducing effect of etoposide and TMZ through downregulation of p53 mRNAs and upregulation of BAX-Bcl-2 in T98G and U87MG cells [39,42,55].

However, a contradictory finding showed that curcumin did not induce cell cycle arrest, as it enhanced cyclin B1 and decreased p21 expression in the radioresistant KNS60 and U251MG(KO) cells [41]. These radioresistant cells usually have a high basal p53 level, but the expression of p53 decreased following curcumin treatment. These results showed that mechanism actions of curcumin in radioresistant GBM cells are different.

### 2.3. JAK/STAT Pathway

Cytokines and growth factors can activate the Janus kinase/signal transducers and activators of transcription (JAK/STAT) pathway to regulate cell proliferation, differentiation, migration, and apoptosis. This pathway involves the activation of growth factor receptor kinases, phosphorylation, dimerization, and translocation of STAT proteins into the nucleus to activate the downstream target genes. JAKs (Jak1, JAK2, JAK3, Tyk2) are the cytoplasmic tyrosine kinases that relay intracellular signals originating from extracellular receptors [77,78,79]. Following JAKs activation and phosphorylation of the tyrosine residues on receptors, STAT is activated through its recruitment and binding to the phosphorylated tyrosine residues. The activation of STATs only lasts from a few minutes to several hours under normal physiological conditions. However, aberrant activation of STAT signaling is found in many GBM tissues compared with normal human astrocytes, white matter, and normal adjacent tissue to the tumor [29,80,81]. Studies showed that the inhibition of either JAK or STAT phosphorylation is associated with reduced levels of anti-apoptotic proteins, resulting in apoptosis in GBM cells [29,43,81,82,83].

As shown in Figure 1c, curcumin can inhibit STAT activation and its downstream target genes involved in cell proliferation, migration, and invasion. Curcumin inhibits JAK1,2/STAT3 tyrosine-phosphorylation, and STAT3 target genes such as *c-Myc*, *MMP-9*, *Snail*, *Twist*, and *Ki67*, which in turn decrease GBM cell migration, invasion, and proliferation [29]. In the same study, curcumin significantly decreased the tumor cell proliferation and growth of the mid-line crossing in the intracranially implanted tumor-bearing mice compared with the control diet. Moreover, curcumin treatment resulted in 15% and 38% tumor-free long-term survival in Tu-2449-bearing mice and Tu-9648-bearing mice, respectively, where all control mice died. Consistently, another study supported that curcumin is capable of inhibiting cell proliferation through the inhibition of STAT3 protein along with reduction *c-Myc* and *Ki-67* transcription in several glioma cell lines [43]. Moreover, curcumin can inhibit the DNMTanalogue M.Sssl to demethylate the *RANK* CpG sites and transcriptionally upregulate *RANK* gene expression in U251 cells [44]. This study further reported that curcumin induces *RANK* expression through STAT3 suppression. Activation of RANK has been known to be associated with pro-apoptotic and anti-tumorigenesis activities [84,85]. Additionally, inhibition of STAT3 can result in suppression of STAT3-DNMT1 interaction, which then demethylates tumor suppressor gene promoters [86]. Other than that, curcumin decreases the Tyr705 and increases the Ser737 phosphorylated form of STAT3 in human patient-derived GSC lines [30]. This causes the inactivation of STAT3 proteins via suppression of its nucleus translocation, which suppresses the activation of its downstream target genes, such as *survivin*, which inhibits GBM cell proliferation.

### 2.4. MAP Kinase Pathway

The mitogen-activated protein kinase (MAPK) pathway is a three-layer signaling cascade. This MAPK cascade is comprised of MAPK3, which activates MAPKK2 through serine/threonine phosphorylation, which then activates MAPK through tyrosine/threonine phosphorylation within a conserved Thr–Xxx–Tyr motif in the activation loop of the kinase domain [87]. There are at least 11 members of the MAPK superfamily, which are divided into three main groups: the extracellular signal-regulated protein kinases (ERK), c-Jun N-terminal kinases (JNK), and p38s [88]. Generally, *ERK* genes are activated by growth factors and mitogen, and the signaling cascades include RAF as MAPKKK and MEK as MAPK. The activation of ERK signaling promotes cell growth, apoptosis, differentiation, and development [89]. While p38s and JNK are activated by stress, inflammatory cytokines, and growth factors [90], their signaling cascades include MEKK as MAPKKK and MKK as MAPKK. Activation of either *p38s* or *JNK* genes may support inflammation, cell apoptosis, cell motility, growth, and chromatin remodeling. ERK, p38, and JNK signaling pathways favor both anti-apoptotic and pro-apoptotic proteins, depending on the cell type and condition [91]. Hence, the aberrant activation or deactivation of this MAPK pathway can promote abnormal cell proliferation, contributing to tumorigenesis.

Studies showed that targeting the MEK-ERK1/2 pathway is one of the approaches to block adhesion of GBM cells onto gelatin/collagen component of ECM, therefore decreasing the proliferation and migration of GBM cells [92,93]. The high expression of p38 had a positive correlation with the glioma’s malignancy grade, while suppressing p38 expression inhibited proliferation and induced apoptosis in GBM cells [94]. Ken-ichiro Matsuda and colleagues discovered that self-renewing stem-like GBM cells have elevated JNK phosphorylation levels, accompanied by increased c-JUN phosphorylation at the cognate JNK phosphorylation site [95]. Treatment with JNK inhibitor reduced the self-renewing ability of the stem-like GBM cells, suggesting JNK is needed for self-renewal in vitro and in vivo. Several studies showed that the inhibition of ERK, JNK, and p38 MAPK pathways induced GBM cell cycle arrest and inhibited cell proliferation [96,97,98,99].

Curcumin can modulate the MAPK signaling pathway to regulate cell proliferation, tumorigenesis, apoptosis, and inflammation, as shown in Figure 1d. So-Young Kim and colleagues discovered that curcumin potently inhibited glioma invasion by inhibiting all the MAPK pathways (JNK, p38, ERK), which then suppressed phorbol myristate acetate (PMA)-induced mRNA expression of *MMP-1, -3, -9,* and *-14* in U87MG and U373MG cells [45,46]. Overexpression of the matrix metalloproteinases (MMPs) facilitates migration and invasion of malignant brain tumor cells to the surrounding brain tissues. These MMPs are upregulated in human malignant gliomas [100]. Among the MMPs, MMP-9 is the most common enzyme that promotes brain tumor invasion and is frequently found in GBM [101]. MMP-1 protein level is increased with the tumor grade and correlated with increased glioma invasiveness [102]. At the same time, the activation of MMP-3 can degrade the brain’s hyaluronic acid-rich matrix, which leads to the invasion and migration of tumor cells [103]. Additionally, MMP-14 is a membrane-bound protease that can remodel the ECM to stimulate proMMP-2 activation. Curcumin treatment (10 µM) inhibits cell invasion by more than 90% in U87MG and U373MG cells [45].

In contrast, curcumin increases the phosphorylated ERK, p38, and c-Jun proteins levels, which decreases GBM stem cell (GSCs) proliferation, sphere-forming ability, and colony-forming potential [30]. Curcumin can also promote MAPK pathway activation through the induction of reactive oxygen species (ROS) [30]. The production of intracellular ROS can induce the activation of ERK and p38 MAPK pathways through oxidative modification of intracellular kinases and inactivation of the MAPK phosphatases [104,105]. Moreover, curcumin induced Egr-1 expression through the activation of p38, ERK, and JNK pathways, which mediated the transactivation of Elk-1 in U87MG human GBM cells [47]. Egr-1 binds directly to the p21 promoter to stimulate p21 transcription, inhibiting CDK activity and resulting in cell cycle arrest. Elk-1 is the direct target gene of the p38, ERK, and JNK pathways, which can form a complex with serum response factor on the serum response element of the Egr-1 promoter to activate the p53-independent transcriptional activation of p21Waf/Cip1 protein. Additionally, curcumin can induce autophagy in GBM cells through the inhibition of the ERK1/2 pathway [48]. Another study suggests that the inhibition of the ERK pathway can lead to suppression of TORC1, which plays an important role in inhibiting autophagy initiation through the phosphorylation of Atg13, ULK, AMBRA, and Atg-14L [106]. In a C6 orthotropic xenograft, curcumin suppressed the phosphorylated JNK1 and JNK2 levels, which decreased lipopolysaccharide-induced CCL2 production [49]. The overexpression of CCL2 can contribute to GBM progression by inducing encephalopathy with mild perivascular leukocyte infiltration, impaired BBB function, and increased expression of proinflammatory cytokine expression [107].

### 2.5. P13K/AKT Pathway

The phosphatidylinositol-3-kinase (P13K)/Akt signaling pathway is critical in regulating cell growth, cell cycle arrest, apoptosis, and mRNA translation to maintain normal physiological conditions. There are three different classes of P13Ks, namely Class I, II, and III, which are categorized accordingly to their different structure and specific substrates [108,109]. Class I P13Ks are heterodimers consisting of a p110 catalytic subunit and p85 adaptor subunit [110,111]. Notably, Class 1 P13Ks is the most common type of P13K, which is incriminated in human cancer. The binding of cytokines or growth factors to the corresponding receptors results in the tyrosine residue autophosphorylation, followed by P13K binding protein recruitment. Upon allosteric activation of the p110 catalytic subunit, P13K catalyzes the phosphorylation of PtdIns(4,5) P2 (PIP2) to PtdIns(3,4,5) P3 (PIP3), which then recruits a subset of signaling proteins with pleckstrin homology (PH), such as AKT and PDK1, to initiate cell proliferation pathways. Protein-phosphatase and tensin homologue (PTEN) act to dephosphorylate PIP3 into PIP2 to prevent activation of the downstream kinases [110,111]. One of Akt’s common target proteins is mTOR, which regulates cell growth and proliferation by promoting biosynthesis of multiple proteins such as cyclin D1, HIF, and VEGF [110]. The mTORC1 activates S6K and inactivates 4EBP1, promoting the production and translation of proteins to promote cell growth. While the mechanism of mTORC2 is less well clarified, it has the same responsibility in promoting cell proliferation [110].

Studies have reported that mutations of the core genes involved in P13K/AKT pathways are commonly found in human GBM tissues [60,112]. Alfeu Zanotto-Flho and colleagues reported that the P13K/Akt pathway are highly upregulated (seven- to eight-fold) in C6 and U138MG cell lines compared to the normal astrocytes cells [33]. Consistently, data from the TCGA pilot project showed that most of the GBM samples acquire homozygous deletion or mutation of *PTEN*, *P13K* mutation, and amplification of *AKT* and *FOXO* genes [60]. Another report indicated that genetic alterations such as loss of heterozygosity (LOH), mutation, and methylation have been identified in most GBM patients. LOH or *PTEN* mutation is positively associated with the poor survival of GBM patients [113]. It was reported that the delivery of Akt small-molecule inhibitor to inhibit the P13K/AKT pathway effectively suppressed the growth of both stem and non-stem GBM cell populations [114].

As seen in Figure 1e, the P13K/Akt signaling pathway and its key molecular targets are inhibited by curcumin to prevent GBM progression. Curcumin inhibits 80% of the P13K/Akt pathway’s constitutive activation by suppressing the phosphorylation of Akt proteins on Ser473 [33]. Inhibition of the P13K/Akt pathway resulted in the induction of G2/M phase arrest as an early step of the apoptotic mechanism, which could probably explain how curcumin spared the non-transformed and its selectivity towards the tumor cells. In the same study, curcumin decreased GBM tumor size and increased apoptotic tumor cells in C6-implanted Wistar rats. Most importantly, curcumin did not cause any tissue toxicity in the rats’ liver, kidney, lungs, or heart. Other than that, curcumin inhibited the P13K/Akt pathway by increasing PTEN expression, which decreased p-Akt and p-mTOR expression, leading to cell apoptosis [31]. In the same study, curcumin also inhibited GBM tumor growth by increasing PTEN protein expression in the U87 xenograft model.

Additionally, curcumin induces autophagy by inhibiting the AKT/mTOR/p70S6K pathway in GBM cell lines and xenograft models [48,50]. In these studies, curcumin significantly decreased the levels of P13Kp85, phosphoP13Kp85, total Akt, p-AKT, mTOR, and p-mTOR. mTOR is not only a major effector of cell growth and proliferation, but it can also inhibit autophagy events in its active form [115]. Thus, inhibiting expression of P13K and AKT, which regulate mTOR expression, is a feasible strategy to induce autophagy–cell death in GBM cells. Notably, curcumin downregulates Bcl-2 and upregulates BAX, leading to the release of cytochrome-c and caspase-3 activation. Curcumin also enhances the anti-cancer effects of nimustine hydrochloride (ACNU) against GBM by inhibiting the phosphorylation of P13K and the AKT (serine/threonine) [51,116].

### 2.6. Sonic Hedgehog (Shh) Pathway

The hedgehog (Hh) signaling pathway is critical for embryonic development, organogenesis, regeneration, and homeostasis for adult tissue [117]. There are three main types of Hh proteins, which are sonic hedgehog (Shh), Indian hedgehog (Ihh), and desert hedgehog (Dhh). The activation of the Shh pathway can occur either through canonical or non-canonical signaling pathways [117]. The canonical Shh activation occurs by ligand-dependent interaction when Shh binds to the patched transmembrane receptor (PTCH) [117,118]. Following this binding, PTCH is incapable of inhibiting the second transmembrane protein, smoothened (Smo). Smo signals the suppressor of fused (SUFU), which is the negative regulator of glioma-associated oncogene homologue (GLI), to release and activate GLI. The activated GLI translocates into the nucleus and modulates downstream gene expression. On the other hand, the non-canonical Shh activation occurs through either GLI-independent mechanisms or Smo-independent mechanisms [117,118]. In the GLI-independent mechanism, Smo is no longer inhibited by the PTCH, and therefore it can stimulate the release of calcium ions from the ER to control the growth of the actin cytoskeleton [118]. In contrast, the Smo-independent mechanism is involved in cyclin B activation to increase cell proliferation and survival [118].

Under normal physiological conditions, the Shh pathway is minimally active in differentiated adult tissue, as it is a highly conserved development pathway. The Shh pathway is frequently associated with GBM tumorigenesis [119,120,121,122]. Studies have reported that most of the GBM patient tissues samples exhibited an aberrant activation of Hh signaling with the presence of GLI1 in both nucleus and cytoplasm [119,123]. Among GLI family members, overexpression of GLI1 is mostly associated with poor prognosis in several cancers, including GBMs [32,124,125,126,127]. GLI1 protein can upregulate several target genes such as *PTCH1, CycD1, MYC, Bcl-2, NANOG*, and *SOX2* to promote cell proliferation, apoptosis, angiogenesis, and stem cell self-renewal [128,129,130]. A study showed that inhibiting GLI1 alone significantly decreases the metabolic activity of GBM cells to reduce chemoresistance [119]. This study also revealed that inhibiting the expression of GLI1 proteins can elevate the nuclear p53 level in U87MG cells. Additionally, the overexpression of Smo is significantly associated with poor prognosis in GBM patients [131]. Smo expression inhibition, which suppresses GBM proliferation, migration, invasion, and tumorigenesis, further supports this observation [132].

As shown in Figure 1f, curcumin is a potent inhibitor of the SHH/GLI signaling pathway by downregulating the Shh, Smo, PTCH, and GLI protein levels and its downstream target genes such as *cyclin D1, Bcl-2, and Foxm-1* [32,52,133]. Curcumin inhibits GBM cell proliferation, colony formation, migration, and induced apoptosis through downregulation of both mRNA and protein levels of SHH/GLI1 signaling (Shh, Smo, and GLI1) in U87 and T98G cells [32]. Curcumin also inhibits GLI nuclear translocation, which deactivates its downstream target genes including *cyclin D1, Bcl-1*, and *Foxm-1*. The combination treatment of curcumin and miR-326 can further reduce the tumor volume and prolong the survival period of U87-bearing mice by inhibiting GLI1 proteins compared with miR-326 or curcumin treatment alone [52]. In the same study, curcumin-treated GBM cells significantly decreased the expression of GLI1 protein, and this observation was enhanced with combination treatment with miR-326. The curcumin and miR-326 treatment also increased the expression of caspase-3 cleaved anti-poly ADP ribose polymerase 1 (PARP-1) caspase-in GBM cells. Simultaneously, the pro-survival proteins BCL-XL, MCL1, and RIP1 were decreased compared to the control and curcumin only-treated GBM cells.

### 2.7. NF-κB Pathway

NF-κB is a family of highly conserved transcription factors that regulate the transcription of various genes involved in cellular activities. There are four members under this NF-κB family: NF-κB1(p50/p105), NF-κB2(p52/p100), Rel-like domain-containing protein A (RelA/p65), and c-rel [134]. They form a dimeric complex (either homodimers or heterodimers) and bind to the specific sequences of DNA called response elements (RE) for the transcription of gene involved in cell proliferation, apoptosis, and inflammatory response [133,135]. NF-κB activation can occur through two major signaling pathways: the canonical and the non-canonical NF-κB signaling pathways [136,137]. The canonical pathway is mediated through the nuclear translocation of p50, RelA, and c-Rel into the nucleus and binding to the targeted DNA sequences. In contrast, the non-canonical NF-κB pathway selectively responds to stimulus and activates p100-sequestered NF-κB members, predominantly via translocation of NF-κB p52 and Rel B into the nucleus. NF-κB members normally bind to the DNA sequences of anti-apoptotic, pro-survival, and immune response genes. Several studies have demonstrated that human GBM cells have aberrant NF-κB activity to maintain their tumorigenic activity [138,139,140,141,142,143].

NF-κB p65 subunit is overexpressed in 81% of 69 samples of GBM and is frequently noted in high-grade compared to low-grade astrocytomas [139]. The constitutive activation of NF-κB p65 is detected in 93% of the GBM cells as compared to normal astrocytes. Studies conducted by Baisakhi et al. showed that inhibition of NF-κB activity resulted in decreased IL-8 transcription, which then inhibited GBM cell invasion and migration [140]. A study conducted by Denise Smith et al. demonstrated that GBM cells that are transfected with short hairpin inhibitory RNAs of RelA and c-Rel for six days displayed reduced tumor growth, signifying the role of RelA and c-Rel in GBM [144]. These studies highlight the importance of inhibiting the overactivation of NF-κB subunits as molecular targets in GBM.

Curcumin modulates the NF-κB pathway to confer the anti-inflammation, anti-proliferation, and apoptotic activity in GBM cells, as shown in Figure 1e. Curcumin can downregulate NF-κB activity by decreasing the expression of anti-apoptotic protein Bcl-xL in GBM cell lines [33]. Reducing the Bcl-xL triggers mitochondrial depolarization, which precedes the losses in mitochondrial membrane integrity. This suggests that curcumin induces mitochondrial-mediated apoptosis in GBM following the inhibition of NF-κB pathways. Other than mitochondrial depolarization, curcumin promotes cell cycle arrest in the G2/M phase prior to cell apoptosis. Most importantly, curcumin acts irrespective of the p53 or PTEN mutational status of the cells. Both PTEN and p53 mutated cells had the same experimental outcomes compared with the wild-type cells after being treated with curcumin. This shows that curcumin exerts p53-independent cell death via inhibition of NF-κB pathways. In the same study, the inhibition of NF-κB by curcumin increases the number of apoptotic cells in tumors, further reducing the tumor size and hemorrhagic areas in C6-implanted Wistar rats. This was in line with other studies showing that curcumin increases the IκB inhibitor proteins and decreases the expression of NF-κB-regulated genes that contribute to GBM chemoresistance [51,53,145]. Curcumin also enhances the anticancer effect of nimustine hydrochloride (ACNU) by suppressing the phosphorylation of IκB, p65, and p50, which then decreases COX-2 expression [51]. Additionally, curcumin’s antiproliferative activity might be facilitated through the downregulation of cyclin D1, since the promoter of cyclin D1 is regulated by NF-κB [54]. In the study conducted by Tzuu-Yuan and colleagues, curcumin increased NF-κB transcription factor inhibition in a concentration-dependent manner in GBM 8401 cells [55].

Curcumin improves the cytotoxic effect of PTX by reducing the phosphorylation of IκB and suppressing NF-κB p65 nuclear translocation to inhibit cell growth in C6 rat glioma cells [38]. Furthermore, curcumin upregulates the pro-apoptotic molecular Smac/Diablo to suppress NF-κB and IAPs (cIAP-1 and cIAP-2), which induces apoptosis [42]. Studies suggest a positive feedback system between NF-κB and IAPs, as IAPs can be upregulated by NF-κB and vice versa [146,147,148]. Hence, downregulation of both NF-κB and IAPs protein might further suppress GBM tumorigenesis.

## 3. Issues of Curcumin Bioavailability and Methods to Overcome Them

Despite the promising anticancer mechanisms, curcumin efficacy is hindered by its low bioavailability. Various studies have reported that very low curcumin concentration was detected in blood, tumors, or extraintestinal tissues [149,150,151], which may be due to the poor absorption, rapid metabolism, chemical instability, and rapid systemic elimination characteristics of curcumin [16]. A study reported that orally-administered curcumin at a dose of 500 mg/kg only had 0.06 µg/mL maximum serum concentration, indicating only 1% oral bioavailability [152]. Due to its chemical structure, curcumin has low solubility in neutral or acidic pH. It is fully protonated, unlike in alkaline conditions where it can be hydrolyzed, especially in the intestinal (pH 6.8). Additionally, rapid metabolism and systemic elimination happen through the formation of glucuronides and sulphates by conjugation in the intestine. Studies reported that free curcumin was undetectable, but curcumin glucuronides and sulphates were highly detected in most of the subjects’ serum samples who had been administered with curcumin, and this indicates rapid metabolism of curcumin [153,154].

The first step of pharmaceutical strategies is to improve curcumin solubility and its absorption to overcome this problem. The incorporation of curcumin in solid dispersion, nanoparticles, micelles, conjugates, liposomes, and phytosomal formulations have increased curcumin’s solubility and absorption rate in GBM cells [155,156,157,158,159,160,161]. Studies showed that curcumin-loaded noisome nanoparticles (CM-NP) can more effectively suppress the viability, proliferation, and migration of GSCs by inducing cell cycle arrest and apoptosis [155]. The CM-NP also efficiently increases ROS-suppression of tumor growth and inhibits monocyte chemoattractant protein 1 (MCP1) to reduce the invasiveness of GSCs compared to curcumin alone. Additionally, rats injected with curcumin-loaded PLGA nanoparticles have significantly smaller tumor size after five days of injection, while the group injected with curcumin alone displayed no significant change [156]. Another in vivo study reported that the combination of antisense-oligonucleotide against miR-21 with curcumin-loaded DP micelle complex reduced the tumor volume more effectively than single therapy curcumin or miR21ASO alone [157].

Additionally, relative to natural curcumin, solid lipid curcumin particles can promote cell death and DNA fragmentation by increasing the levels of caspase-3, Bax, and p53 with downregulation of Bcl-2, c-Myc, and Akt proteins in GBM cell lines [159]. The antibody-conjugated biodegradable polymeric nanoparticles (Mab-PLGA NPs) could enhance the photodynamic efficiency of curcumin on DKMG/EGFRvIII GBM cells compared to curcumin loaded biodegradable polymeric nanoparticles alone (56% vs. 24%) [158]. Furthermore, curcumin analogue induces FBXL2-mediated AR ubiquitination, ROS, lipid peroxidation, and suppression of glutathione peroxidase 4 to inhibit growth of TMZ-sensitive and -resistant GBM in vitro and in vivo [160].

Moreover, curcumin liposomes can significantly improve the anti-tumor effects of curcumin by enhancing the uptake effects, apoptosis effects, and endocytic effects of C6 glioma cells and C6 glioma stem cells. Curcumin liposomes were also shown to inhibit tumor growth and increase the survival period of brain glioma-bearing mice [161]. Other than that, a study showed that curcumin-loaded targeted liposomes cross the BBB two-fold higher than the non-targeted liposomes loaded with curcumin [162]. Curcumin-loaded targeted liposomes can more effectively inhibit GBM tumor growth and increase the survival rate of U87 GBM tumor-bearing mice compared to free curcumin as well as the non-targeted liposome-loaded curcumin [162]. Additionally, curcumin phytosome meriva (CCP) has been shown to improve curcumin bioavailability [163], which then help to activate natural killer cells and mediate elimination of GBM and GBM stem cells [164]. Based on the results of the preclinical studies, the use of the conjugate, nanoparticles, micelles, solid lipid, analogues, liposomes, or phytosomal formulation could certainly be clinically developed to benefit GBM patients.

## 4. Clinical Trials

Currently, there is only one clinical study investigating the curcumin effects on 13 newly diagnosed pre-operative GBM patients [23]. In 2014, this clinical study reported the highest serum and intratumoral concentrations of curcumin detected using the micellar curcumin formulation. It was reported that the intratumoral concentration of curcumin detected might not be sufficient to cause short-term antitumor effects. Still, it might help to control tumor growth in a long-term way. Moreover, intratumoral inorganic phosphate was significantly increased by curcumin. This might indicate increased demand for high-energy phosphates or mitochondrial dysfunction, since inorganic phosphate is used for ATP generation [23]. In addition, the side effects of taking curcumin are significantly less severe than the current chemotherapeutic drugs [23]. Thus, the oral administration of micellar curcumin is relatively safer and well-tolerated. However, this clinical trial only involved a small number of patients with a small dose of curcumin. Further clinical trials should be carried out to strengthen the statistical validity with a larger sample size. In the future, phase I/II clinical trials should be carried out to determine the safety and ideal dosage for GBM treatment, and phase III to IV to examine curcumin’s efficacy and potential side effects with a larger sample population. Other than that, randomized controlled trials can be carried out to avoid bias and provide higher accuracy results.

## 5. Conclusions and Future Perspectives

Taken together, curcumin possesses the ability to modulate various core signaling pathways that are commonly dysregulated in GBM. However, among these signaling pathways, a greater emphasis on Rb and Shh pathways could be of focus for future pre-clinical studies, since the current data are still limited. Additionally, the contradictory findings on curcumin modulation of the p53 pathway warrant future investigation and suggest that curcumin use may be selective against radioresistant GBM tumor. Nevertheless, the combination of curcumin with standard chemotherapeutic drugs mainly results in the modulation of multiple signaling pathways that promote their anti-cancer effects. Curcumin’s ability to modulate the major signaling pathways while promoting the efficacy of standard chemotherapeutic drugs warrants its use as a potential nutraceutical-based adjuvant drug for GBM treatment.

Since the clinical studies of curcumin in GBM patients are lacking, it is worthwhile for future clinical studies to incorporate curcumin as a potential neo-adjuvant in GBM. Although issues such as bioavailability, poor absorption, and rapid systemic elimination may hinder its efficacy, the pre-clinical use of nanodelivery has shown great promise while increasing the efficacy of curcumin and chemotherapeutic drugs. Considering this, a greater emphasis should also be given towards the nanoformulations of curcumin in future clinical studies, in combination with the standard chemotherapeutic drugs. Therefore, the multimodal modulation of signaling pathways via nanoformulation of targeted curcumin delivery that can synergize chemotherapeutic drugs efficacy may provide a clinical perspective in GBM therapy.

## Figures and Tables

**Table 1:** Signaling pathways and mechanism of actions targeted by curcumin in vitro and in vivo against glioblastoma (GBM).

Molecular Pathway	GBM Model (In Vitro or In Vivo)	Pathway Mechanism Targeted by Curcumin OR Mechanism of Actions Induced by Curcumin	References
Rb	DBTRG	Induced G1/S phase arrest by upregulating CDKN2A/p16 and downregulating the expression of RB protein	[28]
P53	DBTRG	Induced G2/M phase arrest by upregulating p21 and downregulating cdc2, followed by increasing of p53 protein	[28]
	U251	Induced G2/M phase arrest by upregulating ING4 expression	[37]
	C6	Induced G0/G1 phase arrest and apoptosis by upregulating p53 and p21Waf/Cip1 protein levels	[38]
	U87MG	Enhanced anticancer effect of ETP and TMZ through downregulation of the p53 protein expression	[39]
	A172	Induced paraptosis by regulating the ER-related miRNAs that interact with the p53-BCL2 pathway	[40]
	KSN60 U251MG (KO)	Decreased p21 expression and increased cyclin B1	[41]
	U87MG	Induced apoptosis through increased BAX:BCL2 ratio via mitochondria-mediated pathway	[42]
JAK/STAT	Tu-2449 Tu-9648 Tu-251	Suppressed cell proliferation by inducing G2/M cell cycle arrest and inhibited cell invasion through downregulation of STAT3 target genes *c-Myc*, *MMP-9*, *Snail* and *Twist*, *Ki67*	[29]
	C6B3F1 mice	Reduced growth and midline crossing of intracranially implanted tumors and proliferation of tumor, increased tumor-free long term survival rate by 15% and 38%	
	A-172 MZ-18 MZ-54 MZ-256 MZ-304	Inhibited cell proliferation, migration, invasion by decreasing expression of phosphorylated STAT3 protein and its target genes *c-Myc* and *Ki67*	[43]
	U251 U87	Induce epigenetic modifications through suppression of STAT3 protein activity, followed by *RANK* promoter methylation along with RANK activation	[44]
	Glio3 Glio9	Induction of intracellular ROS production through downregulation of STAT3 activity	[30]
MAPK	U87MG U373MG CRT-MG	Inhibited invasion of GBM cells through downregulation of MAP kinase pathway along with decreased PMA-induced mRNA expression of *MMP-1*, *-3*, *-9*, and *-4*	[45,46]
	Glio3 Glio9	Decreased GSC viability through induction of P38, ERK, and JNK activity	[30]
	U87MG	Inhibited cell proliferation by upregulating Egr-1 expression through ERK and JNK pathway	[47]
	U87MG U373MG	Induced autophagy through induction of ERK1/2 pathway	[48]
	C6	Inhibited neuroinflammatory effect through inhibition of LPS-induced CCL2 expression via JNK pathway	[49]
	U138MG U87 U373 C6	Induced cell cycle arrest in G2/M phase and apoptosis by inhibiting Akt phosphorylation on Ser473	[33]
	C6 mice	Reduced tumor size and increased number of apoptotic cells in tumor	
P13K/Akt	U251	Inhibited cell proliferation, migration, and invasion by decreasing P13K/mTOR protein expression and restoring PTEN expression	[31]
	U87		
	U87 xenograft	Inhibited tumor growth and increased PTEN expression	
	U87MG GL261 F98 U373MG	Induced autophagy through suppression of AKT/mTOR/p70S6K pathway	[48,50]
	U87 xenograft	Inhibited tumor growth and induced autophagy	
	U118MG U251MG U87MG	Enhanced anti-proliferation, anti-migration, and proapoptotic activities of ACNU against GBM by suppressing P13K/Akt pathway	[51]
Shh	U87 T98G	Inhibited GBM cell proliferation, migration, and invasion through suppressing core components and GLI1-dependent target genes in Shh/GLI1 pathway	[32]
	U87 xenograft	Inhibited GBM growth and prolonged the survival rate	
	U87 U251	Promoted expression of mi-R326 and enhanced inhibition of SHH/GLI1 pathway	[52]
	U87 xenograft	Inhibited GBM growth and prolonged the survival rate	
	U138MG C6	Induced apoptosis through inhibition of NF-κB survival pathways by downregulating the antiapoptotic proteins	[33]
	C6-implanted rats	Decreased brain tumors (growth/size/) without reported tissues, metabolic, oxidative, or hematology toxicity	
NF-κB	U118MG U251MG U887MG	Blocked GBM tumor growth by inhibiting NF-κB/COX-2 pathway	[51]
	T98G	Induced apoptosis through downregulation of NF-κB, IAPs, and upregulation expression of IκBα	[53]
	NP-2	Induced tumor cell death through downregulation of NF-κB activity and its regulated protein cyclin D1	[54]
	GBM 8401	Induced apoptosis through inhibition of NF-κB activity	[55]
	C6	Induced cytotoxic and antiproliferative activity of PTX through inhibition of NF-κB signaling pathway	[38]
	U87MG	Induced apoptosis through suppression of apoptosis protein inhibitor and downregulation of anti-apoptotic NF-κB dependent genes	[42]

## References

[1] Hanif F., Muzaffar K., Perveen K., Malhi S.M., Simjee S.U. (2017). Glioblastoma Multiforme: A Review of its Epidemiology and Pathogenesis through Clinical Presentation and Treatment. Asian Pac. J. Cancer Prev..

[2] Ostrom Q.T., Cioffi G., Gittleman H., Patil N., Waite K., Kruchko C. (2019). CBTRUS Statistical Report: Primary Brain and Other Central Nervous System Tumors Diagnosed in the United States in 2012–2016. Neuro Oncol..

[3] Louis D.N., Perry A., Reifenberger G., Von Deimling A., Figarella-Branger D., Cavenee W.K. (2016). The 2016 World Health Organization Classification of Tumors of the Central Nervous System: A summary. Acta Neuropathol..

[4] Fernandes C., Costa A., Osório L., De Vleeschouwer S. (2017). Current Standards of Care in Glioblastoma Therapy. Glioblastoma [Internet].

[5] Roy S., Lahiri D., Maji T., Biswas J. (2015). Recurrent Glioblastoma: Where we stand. South Asian J. Cancer.

[6] Wilson T.A., Karajannis M.A., Harter D.H. (2014). Glioblastoma multiforme: State of the art and future therapeutics. Surg. Neurol. Int..

[7] Davis M.E. (2016). Glioblastoma: Overview of Disease and Treatment. Clin. J. Oncol. Nurs..

[8] Lee S.Y. (2016). Temozolomide resistance in glioblastoma multiforme. Genes Dis..

[9] Gilbert M.R., Dignam J.J., Armstrong T.S., Wefel J.S., Blumenthal D.T., Vogelbaum M.A. (2014). A Randomized Trial of Bevacizumab for Newly Diagnosed Glioblastoma. N. Engl. J. Med..

[10] Poulsen H.S., Urup T., Michaelsen S.R., Staberg M., Villingshøj M., Lassen U. (2014). The impact of bevacizumab treatment on survival and quality of life in newly diagnosed glioblastoma patients. Cancer Manag. Res..

[11] Chahar M.K., Sharma N., Dobhal M.P., Joshi Y.C. (2011). Flavonoids: A versatile source of anticancer drugs. Pharmacogn. Rev..

[12] Panche A.N., Diwan A.D., Chandra S.R. (2016). Flavonoids: An overview. J. Nutr. Sci..

[13] Hewlings S.J., Kalman D.S. (2017). Curcumin: A Review of Its Effects on Human Health. Foods.

[14] Gupta S.C., Patchva S., Aggarwal B.B. (2013). Therapeutic roles of curcumin: Lessons learned from clinical trials. AAPS J..

[15] Maugeri A., Mazzone M.G., Giuliano F., Vinciguerra M., Basile G., Barchitta M. (2018). Curcumin Modulates DNA Methyltransferase Functions in a Cellular Model of Diabetic Retinopathy. Oxid. Med. Cell. Longev..

[16] Ma Z., Wang N., He H., Tang X. (2019). Pharmaceutical strategies of improving oral systemic bioavailability of curcumin for clinical application. J. Control. Release.

[17] Aggarwal B.B., Harikumar K.B. (2009). Potential therapeutic effects of curcumin, the anti-inflammatory agent, against neurodegenerative, cardiovascular, pulmonary, metabolic, autoimmune and neoplastic diseases. Int. J. Biochem. Cell Biol..

[18] Barchitta M., Maugeri A., Favara G., Magnano San Lio R., Evola G., Agodi A. (2019). Nutrition and Wound Healing: An Overview Focusing on the Beneficial Effects of Curcumin. Int. J. Mol. Sci..

[19] Sharma R.A., McLelland H.R., Hill K.A., Ireson C.R., Euden S.A., Manson M.M. (2001). Pharmacodynamic and Pharmacokinetic Study of Oral Curcuma Extract in Patients with Colorectal Cancer. Clin. Cancer Res..

[20] He Z.-Y., Shi C.-B., Wen H., Li F.-L., Wang B.-L., Wang J. (2011). Upregulation of p53 Expression in Patients with Colorectal Cancer by Administration of Curcumin. Cancer Investig..

[21] Kanai M., Yoshimura K., Asada M., Imaizumi A., Suzuki C., Matsumoto S. (2011). A phase I/II study of gemcitabine-based chemotherapy plus curcumin for patients with gemcitabine-resistant pancreatic cancer. Cancer Chemother. Pharmacol..

[22] Polasa K., Raghuram T.C., Krishna T.P., Krishnaswamy K. (1992). Effect of turmeric on urinary mutagens in smokers. Mutagenesis.

[23] Dützmann S., Schiborr C., Kocher A., Pilatus U., Hattingen E., Weissenberger J. (2016). Intratumoral Concentrations and Effects of Orally Administered Micellar Curcuminoids in Glioblastoma Patients. Nutr. Cancer.

[24] Giordano A., Tommonaro G. (2019). Curcumin and Cancer. Nutrients.

[25] Barati N., Momtazi-Borojeni A.A., Majeed M., Sahebkar A. (2019). Potential therapeutic effects of curcumin in gastric cancer. J. Cell. Physiol..

[26] Hesari A., Azizian M., Sheikhi A., Nesaei A., Sanaei S., Mahinparvar N. (2019). Chemopreventive and therapeutic potential of curcumin in esophageal cancer: Current and future status. Int. J. Cancer.

[27] Jalili-Nik M., Soltani A., Moussavi S., Ghayour-Mobarhan M., Ferns G.A., Hassanian S.M. (2018). Current status and future prospective of Curcumin as a potential therapeutic agent in the treatment of colorectal cancer. J. Cell. Physiol..

[28] Su C.-C., Wang M.-J., Chiu T.-L. (2010). The anti-cancer efficacy of curcumin scrutinized through core signaling pathways in glioblastoma. Int. J. Mol. Med..

[29] Weissenberger J., Priester M., Bernreuther C., Rakel S., Glatzel M., Seifert V. (2010). Dietary Curcumin Attenuates Glioma Growth in a Syngeneic Mouse Model by Inhibition of the JAK1,2/STAT3 Signaling Pathway. Clin. Cancer Res..

[30] Gersey Z.C., Rodriguez G.A., Barbarite E., Sanchez A., Walters W.M., Ohaeto K.C. (2017). Curcumin decreases malignant characteristics of glioblastoma stem cells via induction of reactive oxygen species. BMC Cancer.

[31] Wang Z., Liu F., Liao W., Yu L., Hu Z., Li M. (2020). Curcumin suppresses glioblastoma cell proliferation by p-AKT/mTOR pathway and increases the PTEN expression. Arch. Biochem. Biophys..

[32] Du W.-Z., Feng Y., Wang X.-F., Piao X.-Y., Cui Y.-Q., Chen L.-C. (2013). Curcumin suppresses malignant glioma cells growth and induces apoptosis by inhibition of SHH/GLI1 signaling pathway in vitro and vivo. CNS Neurosci. Ther..

[33] Zanotto-Filho A., Braganhol E., Edelweiss M.I., Behr G.A., Zanin R., Schröder R., Simões-Pires A., Battastini A.M.O., Moreira J.C.F. (2012). The curry spice curcumin selectively inhibits cancer cells growth in vitro and in preclinical model of glioblastoma. J. Nutr. Biochem..

[34] Mao H., Lebrun D.G., Yang J., Zhu V.F., Li M. (2012). Deregulated signaling pathways in glioblastoma multiforme: Molecular mechanisms and therapeutic targets. Cancer Investig..

[35] Chiu S.S., Lui E., Majeed M., Vishwanatha J.K., Ranjan A.P., Maitra A. (2011). Differential Distribution of Intravenous Curcumin Formulations in the Rat Brain. Anticancer Res..

[36] Priyadarsini K.I. (2014). The chemistry of curcumin: From extraction to therapeutic agent. Molecules.

[37] Liu E., Wu J., Cao W., Zhang J., Liu W., Jiang X. (2007). Curcumin induces G2/M cell cycle arrest in a p53-dependent manner and upregulates ING4 expression in human glioma. J. Neuro-Oncol..

[38] Fratantonio D., Molonia M.S., Bashllari R., Muscarà C., Ferlazzo G., Costa G. (2019). Curcumin potentiates the antitumor activity of Paclitaxel in rat glioma C6 cells. Phytomedicine.

[39] Ramachandran C., Nair S.M., Escalon E., Melnick S.J. (2012). Potentiation of Etoposide and Temozolomide Cytotoxicity by Curcumin and Turmeric Force in Brain Tumor Cell Lines. J. Complementary Integr. Med..

[40] Garrido-Armas M., Corona J.C., Escobar M.L., Torres L., Ordóñez-Romero F., Hernández-Hernández A. (2018). Paraptosis in human glioblastoma cell line induced by curcumin. Toxicol. In Vitro.

[41] Khaw A.K., Hande M.P., Kalthur G., Hande M.P. (2013). Curcumin inhibits telomerase and induces telomere shortening and apoptosis in brain tumour cells. J. Cell. Biochem..

[42] Karmakar S., Banik N.L., Ray S.K. (2007). Curcumin Suppressed Anti-apoptotic Signals and Activated Cysteine Proteases for Apoptosis in Human Malignant Glioblastoma U87MG Cells. Neurochem. Res..

[43] Senft C., Polacin M., Priester M., Seifert V., Kögel D., Weissenberger J. (2010). The nontoxic natural compound Curcumin exerts anti-proliferative, anti-migratory, and anti-invasive properties against malignant gliomas. BMC Cancer.

[44] Wu B., Yao X., Nie X., Xu R. (2013). Epigenetic reactivation of RANK in glioblastoma cells by curcumin: Involvement of STAT3 inhibition. DNA Cell Biol..

[45] Kim S.-Y., Jung S.-H., Kim H.-S. (2005). Curcumin is a potent broad spectrum inhibitor of matrix metalloproteinase gene expression in human astroglioma cells. Biochem. Biophys. Res. Commun..

[46] Woo M.-S., Jung S.-H., Kim S.-Y., Hyun J.-W., Ko K.-H., Kim W.-K. (2005). Curcumin suppresses phorbol ester-induced matrix metalloproteinase-9 expression by inhibiting the PKC to MAPK signaling pathways in human astroglioma cells. Biochem. Biophys. Res. Commun..

[47] Choi B.H., Kim C.G., Bae Y.-S., Lim Y., Lee Y.H., Shin S.Y. (2008). p21Waf1/Cip1 Expression by Curcumin in U-87MG Human Glioma Cells: Role of Early Growth Response-1 Expression. Cancer Res..

[48] Aoki H., Takada Y., Kondo S., Sawaya R., Aggarwal B.B., Kondo Y. (2007). Evidence That Curcumin Suppresses the Growth of Malignant Gliomas in Vitro and in Vivo through Induction of Autophagy: Role of Akt and Extracellular Signal-Regulated Kinase Signaling Pathways. Mol. Pharmacol..

[49] Zhang Z.-J., Zhao L.-X., Cao D.-L., Zhang X., Gao Y.-J., Xia C. (2012). Curcumin Inhibits LPS-Induced CCL2 Expression via JNK Pathway in C6 Rat Astrocytoma Cells. Cell. Mol. Neurobiol..

[50] Maiti P., Scott J., Sengupta D., Al-Gharaibeh A., Dunbar G.L. (2019). Curcumin and Solid Lipid Curcumin Particles Induce Autophagy, but Inhibit Mitophagy and the PI3K-Akt/mTOR Pathway in Cultured Glioblastoma Cells. Int. J. Mol. Sci..

[51] Zhao J., Zhu J., Lv X., Xing J., Liu S., Chen C. (2017). Curcumin potentiates the potent antitumor activity of ACNU against glioblastoma by suppressing the PI3K/AKT and NF-κB/COX-2 signaling pathways. OncoTargets Ther..

[52] Yin S., Du W., Wang F., Han B., Cui Y., Yang D. (2018). MicroRNA-326 sensitizes human glioblastoma cells to curcumin via the SHH/GLI1 signaling pathway. Cancer Biol. Ther..

[53] Karmakar S., Banik N.L., Patel S.J., Ray S.K. (2006). Curcumin activated both receptor-mediated and mitochondria-mediated proteolytic pathways for apoptosis in human glioblastoma T98G cells. Neurosci. Lett..

[54] Nagai S., Kurimoto M., Washiyama K., Hirashima Y., Kumanishi T., Endo S. (2005). Inhibition of Cellular Proliferation and Induction of Apoptosis by Curcumin in Human Malignant Astrocytoma Cell Lines. J. Neuro-Oncol..

[55] Huang T.-Y., Tsai T.-H., Hsu C.-W., Hsu Y.-C. (2010). Curcuminoids Suppress the Growth and Induce Apoptosis through Caspase-3-Dependent Pathways in Glioblastoma Multiforme (GBM) 8401 Cells. J. Agric. Food Chem..

[56] Giacinti C., Giordano A. (2006). RB and cell cycle progression. Oncogene.

[57] Knudsen E.S., Wang J.Y.J. (2010). Targeting the RB-pathway in cancer therapy. Clin. Cancer Res..

[58] Nakada M., Kita D., Watanabe T., Hayashi Y., Teng L., Pyko I.V. (2011). Aberrant Signaling Pathways in Glioma. Cancers.

[59] Biernat W., Tohma Y., Yonekawa Y., Kleihues P., Ohgaki H. (1997). Alterations of cell cycle regulatory genes in primary (de novo) and secondary glioblastomas. Acta Neuropathol..

[60] Cancer Genome Atlas Research Network (2008). Comprehensive genomic characterization defines human glioblastoma genes and core pathways. Nature.

[61] Grzmil M., Hemmings B.A. (2010). Deregulated signalling networks in human brain tumours. Biochim. Biophys. Acta BBA Proteins Proteom..

[62] Biasoli D., Kahn S.A., Cornélio T.A., Furtado M., Campanati L., Chneiweiss H. (2013). Retinoblastoma protein regulates the crosstalk between autophagy and apoptosis, and favors glioblastoma resistance to etoposide. Cell Death Dis..

[63] Zhang D., Dai D., Zhou M., Li Z., Wang C., Lu Y. (2018). Inhibition of Cyclin D1 Expression in Human Glioblastoma Cells is Associated with Increased Temozolomide Chemosensitivity. Cell. Physiol. Biochem..

[64] Fry D.W., Harvey P.J., Keller P.R., Elliott W.L., Meade M., Trachet E. (2004). Specific inhibition of cyclin-dependent kinase 4/6 by PD 0332991 and associated antitumor activity in human tumor xenografts. Mol. Cancer Ther..

[65] Michaud K., Solomon D.A., Oermann E., Kim J.-S., Zhong W.-Z., Prados M.D. (2010). Pharmacologic inhibition of cyclin-dependent kinases 4 and 6 arrests the growth of glioblastoma multiforme intracranial xenografts. Cancer Res..

[66] Chen J. (2016). The Cell-Cycle Arrest and Apoptotic Functions of p53 in Tumor Initiation and Progression. Cold Spring Harb. Perspect. Med..

[67] Shangary S., Wang S. (2009). Small-Molecule Inhibitors of the MDM2-p53 Protein-Protein Interaction to Reactivate p53 Function: A Novel Approach for Cancer Therapy. Annu. Rev. Pharmacol. Toxicol..

[68] Kubbutat M.H.G., Jones S.N., Vousden K.H. (1997). Regulation of p53 stability by Mdm2. Nature.

[69] Hemann M.T., Lowe S.W. (2006). The p53-Bcl-2 connection. Cell Death Differ..

[70] Zawlik I., Kita D., Vaccarella S., Mittelbronn M., Franceschi S., Ohgaki H. (2009). Common Polymorphisms in the MDM2 and TP53 Genes and the Relationship between TP53 Mutations and Patient Outcomes in Glioblastomas. Brain Pathol..

[71] Fels C., Schäfer C., Hüppe B., Bahn H., Heidecke V., Kramm C.M. (2000). Bcl-2 Expression in Higher-grade Human Glioma: A Clinical and Experimental Study. J. Neuro-Oncol..

[72] Zhang Y., Dube C., Gibert M., Cruickshanks N., Wang B., Coughlan M. (2018). The p53 Pathway in Glioblastoma. Cancers.

[73] Ghaemi S., Arefian E., Rezazadeh Valojerdi R., Soleimani M., Moradimotlagh A., Jamshidi Adegani F. (2020). Inhibiting the expression of anti-apoptotic genes BCL2L1 and MCL1, and apoptosis induction in glioblastoma cells by microRNA-342. Biomed. Pharmacother..

[74] Pareja F., Macleod D., Shu C., Crary J.F., Canoll P.D., Ross A.H. (2014). PI3K and Bcl-2 Inhibition Primes Glioblastoma Cells to Apoptosis through Downregulation of Mcl-1 and Phospho-BAD. Mol. Cancer Res..

[75] Doyon Y., Cayrou C., Ullah M., Landry A.-J., Côté V., Selleck W. (2006). ING Tumor Suppressor Proteins Are Critical Regulators of Chromatin Acetylation Required for Genome Expression and Perpetuation. Mol. Cell.

[76] Kim S. (2005). HuntING4 New Tumor Suppressors. Cell Cycle.

[77] Yamaoka K., Saharinen P., Pesu M., Holt V.E.T., Silvennoinen O., O’Shea J.J. (2004). The Janus kinases (Jaks). Genome Biol..

[78] Zhou Y.-J., Chen M., Cusack N.A., Kimmel L.H., Magnuson K.S., Boyd J.G. (2001). Unexpected Effects of FERM Domain Mutations on Catalytic Activity of Jak3: Structural Implication for Janus Kinases. Mol. Cell.

[79] Rawlings J.S., Rosler K.M., Harrison D.A. (2004). The JAK/STAT signaling pathway. J. Cell Sci..

[80] Schaefer L.K., Ren Z., Fuller G.N., Schaefer T.S. (2002). Constitutive activation of Stat3α in brain tumors: Localization to tumor endothelial cells and activation by the endothelial tyrosine kinase receptor (VEGFR-2). Oncogene.

[81] Rahaman S.O., Harbor P.C., Chernova O., Barnett G.H., Vogelbaum M.A., Haque S.J. (2002). Inhibition of constitutively active Stat3 suppresses proliferation and induces apoptosis in glioblastoma multiforme cells. Oncogene.

[82] Zhang L., Alizadeh D., Van Handel M., Kortylewski M., Yu H., Badie B. (2009). Stat3 inhibition activates tumor macrophages and abrogates glioma growth in mice. Glia.

[83] Kim H.Y., Park E.J., Joe E.-h., Jou I. (2003). Curcumin Suppresses Janus Kinase-STAT Inflammatory Signaling through Activation of Src Homology 2 Domain-Containing Tyrosine Phosphatase 2 in Brain Microglia. J. Immunol..

[84] Papanastasiou A.D., Sirinian C., Kalofonos H.P. (2012). Identification of novel human receptor activator of nuclear factor-kB isoforms generated through alternative splicing: Implications in breast cancer cell survival and migration. Breast Cancer Res..

[85] Von dem Knesebeck A., Felsberg J., Waha A., Hartmann W., Scheffler B., Glas M. (2012). RANK (TNFRSF11A) is epigenetically inactivated and induces apoptosis in gliomas. Neoplasia.

[86] Lee H., Zhang P., Herrmann A., Yang C., Xin H., Wang Z. (2012). Acetylated STAT3 is crucial for methylation of tumor-suppressor gene promoters and inhibition by resveratrol results in demethylation. Proc. Natl. Acad. Sci. USA.

[87] Soares-Silva M., Diniz F.F., Gomes G.N., Bahia D. (2016). The Mitogen-Activated Protein Kinase (MAPK) Pathway: Role in Immune Evasion by Trypanosomatids. Front. Microbiol..

[88] Teramoto H., Gutkind J.S., Lennarz W.J., Lane M.D. (2013). Mitogen-Activated Protein Kinase Family. Encyclopedia of Biological Chemistry.

[89] Mebratu Y., Tesfaigzi Y. (2009). How ERK1/2 activation controls cell proliferation and cell death: Is subcellular localization the answer?. Cell Cycle.

[90] Huang G., Shi L.Z., Chi H. (2009). Regulation of JNK and p38 MAPK in the immune system: Signal integration, propagation and termination. Cytokine.

[91] Vo V.A., Lee J.-W., Lee H.J., Chun W., Lim S.Y., Kim S.-S. (2014). Inhibition of JNK Potentiates Temozolomide-induced Cytotoxicity in U87MG Glioblastoma Cells via Suppression of Akt Phosphorylation. Anticancer Res..

[92] Ramaswamy P., Nanjaiah N.D., Borkotokey M. (2019). Role of MEK-ERK signaling mediated adhesion of glioma cells to extra-cellular matrix: Possible implication on migration and proliferation. Ann. Neurosci..

[93] Lo H.-W. (2010). Targeting Ras-RAF-ERK and its interactive pathways as a novel therapy for malignant gliomas. Curr. Cancer Drug Targets.

[94] Yang K., Liu Y., Liu Z., Liu J., Liu X., Chen X. (2013). p38γ overexpression in gliomas and its role in proliferation and apoptosis. Sci. Rep..

[95] Matsuda K.-I., Sato A., Okada M., Shibuya K., Seino S., Suzuki K. (2012). Targeting JNK for therapeutic depletion of stem-like glioblastoma cells. Sci. Rep..

[96] Ke X.-X., Pang Y., Chen K., Zhang D., Wang F., Zhu S. (2018). Knockdown of arsenic resistance protein 2 inhibits human glioblastoma cell proliferation through the MAPK/ERK pathway. Oncol. Rep..

[97] Ouyang Z., Xu G. (2019). Antitumor effects of Sweroside in human glioblastoma: Its effects on mitochondrial mediated apoptosis, activation of different caspases, G0/G1 cell cycle arrest and targeting JNK/p38 MAPK signal pathways. J. BUON.

[98] Heiland D.H., Haaker G., Delev D., Mercas B., Masalha W., Heynckes S. (2017). Comprehensive analysis of PD-L1 expression in glioblastoma multiforme. Oncotarget.

[99] Wurm J., Behringer S.P., Ravi V.M., Joseph K., Neidert N., Maier J.P. (2019). Astrogliosis Releases Pro-Oncogenic Chitinase 3-Like 1 Causing MAPK Signaling in Glioblastoma. Cancers.

[100] Munaut C., Noël A., Hougrand O., Foidart J.-M., Boniver J., Deprez M. (2003). Vascular endothelial growth factor expression correlates with matrix metalloproteinases MT1-MMP, MMP-2 and MMP-9 in human glioblastomas. Int. J. Cancer.

[101] Sawaya R., Go Y., Kyritisis A.P., Uhm J., Venkaiah B., Mohanam S. (1998). Elevated Levels of Mr92,000 Type IV Collagenase during Tumor Growthin Vivo. Biochem. Biophys. Res. Commun..

[102] Lorenzl S., Albers D.S., Chirichigno J.W., Augood S.J., Beal M.F. (2004). Elevated levels of matrix metalloproteinases-9 and -1 and of tissue inhibitors of MMPs, TIMP-1 and TIMP-2 in postmortem brain tissue of progressive supranuclear palsy. J. Neurol. Sci..

[103] Bignami A., Hosley M., Dahl D. (1993). Hyaluronic acid and hyaluronic acid-binding proteins in brain extracellular matrix. Anat. Embryol..

[104] Keshari R.S., Verma A., Barthwal M.K., Dikshit M. (2013). Reactive oxygen species-induced activation of ERK and p38 MAPK mediates PMA-induced NETs release from human neutrophils. J. Cell. Biochem..

[105] McCubrey J.A., LaHair M.M., Franklin R.A. (2006). Reactive Oxygen Species-Induced Activation of the MAP Kinase Signaling Pathways. Antioxid. Redox Signal..

[106] Escamilla-Ramírez A., Castillo-Rodríguez R.A., Zavala-Vega S., Jimenez-Farfan D., Anaya-Rubio I., Briseño E. (2020). Autophagy as a Potential Therapy for Malignant Glioma. Pharmaceuticals.

[107] Chang A.L., Miska J., Wainwright D.A., Dey M., Rivetta C.V., Yu D. (2016). CCL2 Produced by the Glioma Microenvironment Is Essential for the Recruitment of Regulatory T Cells and Myeloid-Derived Suppressor Cells. Cancer Res..

[108] Engelman J.A., Luo J., Cantley L.C. (2006). The evolution of phosphatidylinositol 3-kinases as regulators of growth and metabolism. Nat. Rev. Genet..

[109] Katso R., Okkenhaug K., Ahmadi K., White S., Timms J., Waterfield M.D. (2001). Cellular Function of Phosphoinositide 3-Kinases: Implications for Development, Immunity, Homeostasis, and Cancer. Annu. Rev. Cell Dev. Biol..

[110] Porta C., Paglino C., Mosca A. (2014). Targeting PI3K/Akt/mTOR Signaling in Cancer. Front. Oncol..

[111] Yang J., Nie J., Ma X., Wei Y., Peng Y., Wei X. (2019). Targeting PI3K in cancer: Mechanisms and advances in clinical trials. Mol. Cancer.

[112] Brennan C.W., Verhaak R.G.W., McKenna A., Campos B., Noushmehr H., Salama S.R. (2013). The somatic genomic landscape of glioblastoma. Cell.

[113] Koul D. (2008). PTEN Signaling pathways in glioblastoma. Cancer Biol. Ther..

[114] Gallia G.L., Tyler B.M., Hann C.L., Siu I.M., Giranda V.L., Vescovi A.L. (2009). Inhibition of Akt inhibits growth of glioblastoma and glioblastoma stem-like cells. Mol. Cancer Ther..

[115] Kim Y.C., Guan K.-L. (2015). mTOR: A pharmacologic target for autophagy regulation. J. Clin. Investig..

[116] Bava S.V., Puliyappadamba V.T., Deepti A., Nair A., Karunagaran D., Anto R.J. (2018). Sensitization of taxol-induced apoptosis by curcumin involves down-regulation of nuclear factor-κB and the serine/threonine kinase Akt and is independent of tubulin polymerization. J. Biol. Chem..

[117] Carballo G.B., Honorato J.R., De Lopes G.P.F. (2018). A highlight on Sonic hedgehog pathway. Cell Commun. Signal..

[118] Robbins D.J., Fei D.L., Riobo N.A. (2012). The Hedgehog signal transduction network. Sci. Signal..

[119] Melamed J.R., Morgan J.T., Ioele S.A., Gleghorn J.P., Sims-Mourtada J., Day E.S. (2018). Investigating the role of Hedgehog/GLI1 signaling in glioblastoma cell response to temozolomide. Oncotarget.

[120] Ulasov I.V., Nandi S., Dey M., Sonabend A.M., Lesniak M.S. (2011). Inhibition of Sonic hedgehog and Notch pathways enhances sensitivity of CD133(+) glioma stem cells to temozolomide therapy. Mol. Med..

[121] Takezaki T., Hide T., Takanaga H., Nakamura H., Kuratsu J.-I., Kondo T. (2011). Essential role of the Hedgehog signaling pathway in human glioma-initiating cells. Cancer Sci..

[122] Takebe N., Miele L., Harris P.J., Jeong W., Bando H., Kahn M. (2015). Targeting Notch, Hedgehog, and Wnt pathways in cancer stem cells: Clinical update. Nat. Rev. Clin. Oncol..

[123] Rossi M., Magnoni L., Miracco C., Mori E., Tosi P., Pirtoli L. (2011). β-catenin and Gli1 are prognostic markers in glioblastoma. Cancer Biol. Ther..

[124] Honorato J.R., Hauser-Davis R.A., Saggioro E.M., Correia F.V., Sales-Junior S.F., Soares L.O.S. (2020). Role of Sonic hedgehog signaling in cell cycle, oxidative stress, and autophagy of temozolomide resistant glioblastoma. J. Cell. Physiol..

[125] Zhou A., Lin K., Zhang S., Ma L., Xue J., Morris S.-A. (2017). Gli1-induced deubiquitinase USP48 aids glioblastoma tumorigenesis by stabilizing Gli1. EMBO Rep..

[126] Ruiz i Altaba A., Stecca B., Sánchez P. (2004). Hedgehog–Gli signaling in brain tumors: Stem cells and paradevelopmental programs in cancer. Cancer Lett..

[127] Cheng J., Gao J., Tao K. (2016). Prognostic role of Gli1 expression in solid malignancies: A meta-analysis. Sci. Rep..

[128] Hui C.-C., Angers S. (2011). Gli Proteins in Development and Disease. Annu. Rev. Cell Dev. Biol..

[129] Scales S.J., De Sauvage F.J. (2009). Mechanisms of Hedgehog pathway activation in cancer and implications for therapy. Trends Pharmacol. Sci..

[130] Stecca B., Ruiz i Altaba A. (2010). Context-dependent regulation of the GLI code in cancer by HEDGEHOG and non-HEDGEHOG signals. J. Mol. Cell Biol..

[131] Tu Y., Niu M., Xie P., Yue C., Liu N., Qi Z. (2017). Smoothened is a poor prognosis factor and a potential therapeutic target in glioma. Sci. Rep..

[132] Jeng K.-S., Sheen I.S., Leu C.-M., Tseng P.-H., Chang C.-F. (2020). The Role of Smoothened in Cancer. Int. J. Mol. Sci..

[133] Elamin M.H., Shinwari Z., Hendrayani S.-F., Al-Hindi H., Al-Shail E., Khafaga Y. (2010). Curcumin inhibits the Sonic Hedgehog signaling pathway and triggers apoptosis in medulloblastoma cells. Mol. Carcinog..

[134] Puliyappadamba V.T., Hatanpaa K.J., Chakraborty S., Habib A.A. (2014). The role of NF-κB in the pathogenesis of glioma. Mol. Cell. Oncol..

[135] Mitchell S., Vargas J., Hoffmann A. (2016). Signaling via the NFκB system. Wiley Interdiscip. Rev. Syst. Biol. Med..

[136] Xia Y., Shen S., Verma I.M. (2014). NF-κB, an active player in human cancers. Cancer Immunol. Res..

[137] Sun S.-C. (2017). The non-canonical NF-κB pathway in immunity and inflammation. Nat. Rev. Immunol..

[138] Shoichi N., Kazuo W., Masanori K., Akira T., Shunro E., Toshiro K. (2002). Aberrant nuclear factor-κB activity and its participation in the growth of human malignant astrocytoma. J. Neurosurg..

[139] Wang H., Wang H., Zhang W., Huang H.J., Liao W.S.L., Fuller G.N. (2004). Analysis of the activation status of Akt, NFκB, and Stat3 in human diffuse gliomas. Lab. Investig..

[140] Raychaudhuri B., Han Y., Lu T., Vogelbaum M.A. (2007). Aberrant constitutive activation of nuclear factor κB in glioblastoma multiforme drives invasive phenotype. J. Neuro-Oncol..

[141] Atkinson G.P., Nozell S.E., Harrison D.K., Stonecypher M.S., Chen D., Benveniste E.N. (2009). The prolyl isomerase Pin1 regulates the NF-kappaB signaling pathway and interleukin-8 expression in glioblastoma. Oncogene.

[142] Kim S.-H., Ezhilarasan R., Phillips E., Gallego-Perez D., Sparks A., Taylor D. (2016). Serine/Threonine Kinase MLK4 Determines Mesenchymal Identity in Glioma Stem Cells in an NF-κB-dependent Manner. Cancer Cell.

[143] Xu R.X., Liu R.Y., Wu C.M., Zhao Y.S., Li Y., Yao Y.Q. (2015). DNA Damage-Induced NF-κB Activation in Human Glioblastoma Cells Promotes miR-181b Expression and Cell Proliferation. Cell. Physiol. Biochem..

[144] Smith D., Shimamura T., Barbera S., Bejcek B.E. (2008). NF-κB controls growth of glioblastomas/astrocytomas. Mol. Cell. Biochem..

[145] Jiang Z., Zheng X., Rich K.M. (2003). Down-regulation of Bcl-2 and Bcl-xL expression with bispecific antisense treatment in glioblastoma cell lines induce cell death. J. Neurochem..

[146] Mitsiades N., Mitsiades C.S., Poulaki V., Chauhan D., Richardson P.G., Hideshima T. (2002). Biologic sequelae of nuclear factor–κB blockade in multiple myeloma: Therapeutic applications. Blood.

[147] Zou T., Rao J.N., Guo X., Liu L., Zhang H.M., Strauch E.D. (2004). NF-κB-mediated IAP expression induces resistance of intestinal epithelial cells to apoptosis after polyamine depletion. Am. J. Physiol. Cell Physiol..

[148] Jeremias I., Kupatt C., Baumann B., Herr I., Wirth T., Debatin K.M. (1998). Inhibition of Nuclear Factor κB Activation Attenuates Apoptosis Resistance in Lymphoid Cells. Blood.

[149] Kakran M., Sahoo N.G., Tan I.-L., Li L. (2012). Preparation of nanoparticles of poorly water-soluble antioxidant curcumin by antisolvent precipitation methods. J. Nanopart. Res..

[150] Dubey S.K., Sharma A.K., Narain U., Misra K., Pati U. (2008). Design, synthesis and characterization of some bioactive conjugates of curcumin with glycine, glutamic acid, valine and demethylenated piperic acid and study of their antimicrobial and antiproliferative properties. Eur. J. Med. Chem..

[151] Lao C.D., Ruffin M.T., Normolle D., Heath D.D., Murray S.I., Bailey J.M., Boggs M.E., Crowell J., Rock C.L., Brenner D.E. (2006). Dose escalation of a curcuminoid formulation. BMC Complementary Altern. Med..

[152] Tapal A., Tiku P.K. (2012). Complexation of curcumin with soy protein isolate and its implications on solubility and stability of curcumin. Food Chem..

[153] Noguchi-Shinohara M., Hamaguchi T., Yamada M., Farooqui T., Farooqui A.A. (2019). Chapter 5—The Potential Role of Curcumin in Treatment and Prevention for Neurological Disorders. Curcumin for Neurological and Psychiatric Disorders.

[154] Dhillon N., Aggarwal B.B., Newman R.A., Wolff R.A., Kunnumakkara A.B., Abbruzzese J.L. (2008). Phase II Trial of Curcumin in Patients with Advanced Pancreatic Cancer. Clin. Cancer Res..

[155] Sahab-Negah S., Ariakia F., Jalili-Nik M., Afshari A.R., Salehi S., Samini F. (2020). Curcumin Loaded in Niosomal Nanoparticles Improved the Anti-tumor Effects of Free Curcumin on Glioblastoma Stem-like Cells: An In Vitro Study. Mol. Neurobiol..

[156] Orunoğlu M., Kaffashi A., Pehlivan S.B., Şahin S., Söylemezoğlu F., Oğuz K.K. (2017). Effects of curcumin-loaded PLGA nanoparticles on the RG2 rat glioma model. Mater. Sci. Eng. C.

[157] Tan X., Kim G., Lee D., Oh J., Kim M., Piao C. (2018). A curcumin-loaded polymeric micelle as a carrier of a microRNA-21 antisense-oligonucleotide for enhanced anti-tumor effects in a glioblastoma animal model. Biomater. Sci..

[158] Jamali Z., Khoobi M., Hejazi S.M., Eivazi N., Abdolahpour S., Imanparast F. (2018). Evaluation of targeted curcumin (CUR) loaded PLGA nanoparticles for in vitro photodynamic therapy on human glioblastoma cell line. Photodiagn. Photodyn. Ther..

[159] Maiti P., Al-Gharaibeh A., Kolli N., Dunbar G.L. (2017). Solid Lipid Curcumin Particles Induce More DNA Fragmentation and Cell Death in Cultured Human Glioblastoma Cells than Does Natural Curcumin. Oxid. Med. Cell. Longev..

[160] Chen T.-C., Chuang J.-Y., Ko C.-Y., Kao T.-J., Yang P.-Y., Yu C.-H. (2020). AR ubiquitination induced by the curcumin analog suppresses growth of temozolomide-resistant glioblastoma through disrupting GPX4-Mediated redox homeostasis. Redox Biol..

[161] Wang Y., Ying X., Xu H., Yan H., Li X., Tang H. (2017). The functional curcumin liposomes induce apoptosis in C6 glioblastoma cells and C6 glioblastoma stem cells in vitro and in animals. Int. J. Nanomed..

[162] Gabay M., Weizman A., Zeineh N., Kahana M., Obeid F., Allon N. (2020). Liposomal Carrier Conjugated to APP-Derived Peptide for Brain Cancer Treatment. Cell. Mol. Neurobiol..

[163] Mirzaei H., Shakeri A., Rashidi B., Jalili A., Banikazemi Z., Sahebkar A. (2017). Phytosomal curcumin: A review of pharmacokinetic, experimental and clinical studies. Biomed. Pharmacother..

[164] Mukherjee S., Fried A., Hussaini R., White R., Baidoo J., Yalamanchi S. (2018). Phytosomal curcumin causes natural killer cell-dependent repolarization of glioblastoma (GBM) tumor-associated microglia/macrophages and elimination of GBM and GBM stem cells. J. Exp. Clin. Cancer Res..

